# Meta-analysis of the rs231775 locus polymorphism in the CTLA-4 gene and the susceptibility to Graves’ disease in children

**DOI:** 10.1515/biol-2022-0589

**Published:** 2023-04-28

**Authors:** Mili Shi, Xiaobo Liu, Qiuyan Song, Yijie Shi, Qirui Tai

**Affiliations:** Department of Pharmacy, People’s Hospital of Yuxi City, Yuxi, Yunnan 653100, China; College of Pharmacy and Chemistry, Dali University, Dali, Yunnan 671000, China

**Keywords:** CLTA-4, gene polymorphism, Graves’ disease, meta-analysis

## Abstract

The aim of this study was to systematically evaluate the correlation between the rs231775 locus polymorphism in the cytotoxic T lymphocyte-associated antigen 4 (CTLA-4) gene and genetic susceptibility to Graves’ disease (GD) in children. Some studies found that the CTLA-4 gene polymorphism was associated with GD in children. The data up to February 2022 were retrieved from the databases. Stata 15.0 software was used for meta-analysis. A total of seven studies were included in our research. The results of the meta-analysis showed that the rs231775 locus polymorphism in the CTLA-4 gene in general and Asian populations was correlated with children’s susceptibility to GD (A vs G: OR = 0.75, 95% CI (0.660–0.86); GG vs AA: OR = 1.34, 95% CI (1.04–1.73); AG vs AA: OR = 1.32, 95% CI (1.02–1.10); AG + GG vs AA: OR = 3.81, 95% CI (2.17–6.70); GG vs AA + AG: OR *=* 1.23, 95% CI (1.05–1.45)). In summary, the rs231775 locus polymorphism in the CLTA-4 gene may be a risk factor for GD in Asian children. The G allele may be a susceptibility factor, while the allele A may be a protective factor against GD in Asian children. In the future, more large-scale studies may be needed to verify our results.

## 1 Introduction

Graves’ disease (GD) is an autoimmune thyroid disease characterized by TSH receptor antibodies that stimulate the thyroid gland and raise thyroid hormone levels. The development of GD is thought to be determined by genetic and environmental factors, and in terms of genetic factors, it has shown a gradual increase in recent years. Also, it is becoming a common disease [1,2]. It threatens the health of children. Although the occurrence of Graves’ disease is determined by both genetic and environmental factors (infection and stress), a twin study has shown that approximately 80% of susceptibility to GD is caused by genetic factors [3]. The pathogenesis of GD is related to the 49th A/G site at the rs231775 exon 1 locus of the CTLA-4 gene [4,5] in the chromosome 2q33 region. Furthermore, the association between the polymorphism of the rs231775 locus in the CTLA-4 gene and the incidence of GD in adults has been confirmed in GD patients of various ethnicities, including Caucasians [6,7,8,9], Japanese [10], and other ethnic origins [11,12]. The pathogenesis of GD in children differs from that observed in adults, with different research results. Relevant research studying CTLA-4 gene polymorphism and susceptibility to GD in children has concluded inconsistent results between different regions, races, and even between the same regions [4,5,12]. To draw a more accurate conclusion, we used a meta-analysis to systematically assess the correlation between rs231775 locus polymorphism of the CTLA-4 gene and the susceptibility to GD in children. Therefore, our objective was to quantitatively evaluate the impact of the CTLA-4 + 49 A/G polymorphism on the risk factor for GD.

## 2 Data and methods

### 2.1 Retrieval strategy

We searched PubMed, Embase CBM, Cochrane Library, cnki.net, Wanfang Data, and VIP databases with words such as “CTLA-4, gene polymorphism, hyperthyroidism, GD, children, and correlation.” The search period included data from the time the database was established to February 2022. The databases were searched for free words and subject words. After multiple pre-retrieval, the search strategy was confirmed.

### 2.2 Research registration

The present systematic review and meta-analysis were carried out according to the established protocol (PROSPERO: CRD42022343849) (Tai QR et al., 2022). (Available from: https://www.crd.york.ac.uk/prospero/#recordDetails).

### 2.3 Inclusion criteria

(1) Study on the relationship between cytotoxic T lymphocyte-associated antigen-4 (CTLA-4) gene polymorphism and genetic susceptibility (GD) to hyperthyroidism (limited to Chinese and English languages); (2) Patients with a confirmed diagnosis of GD and under 18 years of age; (3) The genotype frequency of the control group determined according to the Hardy–Weinberg equilibrium.

### 2.4 Exclusion criteria

(1) Study including review, animal experiments, conference papers, and *in vitro* cell experiments; (2) Study on the relationship between gene polymorphism at a different locus and GD; (3) Non-case-control study; (4) Incomplete original data; (5) Studies not having direct access to patient age, sample size, and study data.

### 2.5 Data acquisition of literature and quality evaluation methods

Two staff members performed data extraction and quality evaluation along with further cross-verification. A third researcher was also added to the discussion, who solved the issues in case of differences. After the literature selection, the extracted data included the first author, publication year, sample size, age, and specific distribution of the genotypes (AA, AG, GG, A, G) in both the case and control groups. Hardy–Weinberg (H–W) genetic balance test was performed to distribute genotypes between the control groups.

### 2.6 Quality evaluation

As this meta-analysis included only case-control studies, the quality of the literature was scored according to the Newcastle-Ottawa Scale (NOS), which evaluated case-control studies by three blocks with eight items, including study population selection, comparability, and exposure assessment. The NOS score ranged from 0 to 9, with NOS score ≥ 7 as high quality and NOS score < 7 as low quality. Two reviewers evaluated the NOS score to assess the risk of bias in the included studies. In case of disagreement, it was resolved by discussion or by a third party [13].

### 2.7 Statistical analysis

(1) The H–W equilibrium test was adopted to distribute genotypes in the study. (2) Stata 15.0 software was applied for the meta-analysis. Heterogeneity between studies was evaluated using the Cochran Q test. The obvious heterogeneity was found in the data from the published literature with *P* < 0.05, while the heterogeneity was quantitatively evaluated using the *I* ^2^ value. The result of the heterogeneity test at *P* < 0.05 or *I* ^2^ > 50% indicated a great heterogeneity among the studies. Thus, the heterogeneity hypothesis should be explored in a series of sensitivity analyses adopting a random effect model. If this strategy is not used, a fixed-effect model may be applied. (3) The Mantel–Haenszel (M–H) method was used to calculate the combined OR value and its 95% CI. A 95% CI value of less than 1 indicated a significant difference between the case and control groups. (4) The inverted funnel diagram and Begg’s and Egger’s tests were used to determine any publication bias. *P* ≥ 0.05 indicated that there was no publication bias. Furthermore, the trim-and-fill method was used to test and correct its possibility. A 95% CI value of less than 1 indicated that there was no publication bias.

## 3 Results

### 3.1 Evaluation of bias

According to the NOS evaluation scale, the literature scores were above 7, which could be included in this systematic review. The details are displayed in Table 1.

**Table 1:** Basic characteristics of the included studies

Included study	Study area	Race	*N* (case/control)	Case					Control					H–W		NOS score
				AA	AG	GG	A	G	AA	AG	GG	A	G	*X* ^2^	*P*	
Pastuszak-Lewandoska et al., 2013 [17]	Poland	Caucasian	93 (24/69)	3	9	12	15	33	29	27	13	85	53	2.063	0.356	8
Iwama et al., 2005 [4]	Japan	Asian	243 (43/200)	1	25	17	27	59	34	88	78	156	244	1.132	0.568	7
Mochizuki et al., 2003 [22]	Japan	Asian	76 (16/60)	1	5	10	7	25	12	27	21	51	69	0.866	0.649	7
Chong et al., 2018 [23]	Hong Kong, China	Asian	328 (177/151)	7	73	97	87	267	24	56	71	104	198	4.82	0.09	7
Ting et al., 2016 [24]	Mainland, China	Asian	1,323 (265/1,058)	19	97	149	135	395	112	469	477	693	1,423	0.043	0.97	8
Yung et al., 2002 [12]	Hong Kong, China	Asian	281 (123/158)	3	54	66	60	186	23	59	76	105	211	3.96	0.138	8
Cury et al., 2008 [5]	Brazil	Caucasian	122 (44/78)	17	22	5	56	32	39	32	7	110	46	0.014	0.993	8

Note: *P* ≥ 0.05 indicates that the gene conforms to the Hardy–Weinberg equilibrium.

### 3.2 Retrieval results and general characteristics of the included literature

A total of 201 studies were retrieved, including 10 in Chinese and 105 in English. Finally, seven studies (all in English) that met the inclusion criteria were selected for meta-analysis. The included literature belonged to mainland China, Hongkong, Japan, Poland, Brazil, and five other countries and regions. In each study, the genes in the control group followed the H–W equilibrium, while the test values of each group were found to be *P* ≥ 0.05, (Figure S1 and Table 1).

### 3.3 Results of the meta-analysis

The allele model used was A vs G; co-dominant model: GG vs AA and GA vs AA; recessive genetic model: GG vs AG + AA; and super dominant model: GG + AA vs AG. Since the heterogeneity test among the studies did not show a statistically significant difference (*I* ^2^ < 50%, *P* > 0.05), the fixed effect model was adopted. The dominant model was AG + GG vs AA. Since statistically significant differences were observed in the heterogeneity test among studies (*I* ^2^ = 86.0%, *P* < 0.05), the random effect model was applied, as shown in Table 2.

**Table 2:** Meta-analysis of the polymorphism of the rs231775 locus in the CTLA-4 gene and susceptibility to GD in children

Gene model		Heterogeneity test		OR (95% CI)	Post trim-and-fill	Egger’s test		Begg’s test	
		*I* ^2^%	*P*		OR value (95% CI)	*t* value	*P* value	*t* value	*P* value
A vs G	Allele model	0.00	0.782	0.75 (0.66–0.86)*	0.741 (0.649–0.846)	−0.92	0.4	0.548	0.6
GG vs AA	Co-dominant model	0.00	0.852	1.34 (1.04–1.73)*	1.929 (1.026–1.626)	1.05	0.353	0.38	0.707
AG vs AA	Co-dominant model	0.00	1.000	1.32 (1.02–1.10)*	1.288 (1.019–1.628)	0.21	0.848	−0.19	1
AG + GG vs AA	Dominant Model	80.6	0.000	3.81 (2.17–6.70)*	1.177 (0.990–1.400)	0.6	0.582	0.38	0.707
GG vs AA + AG	Recessive model	0.00	0.672	1.23 (1.05–1.45)*	1.249 (1.067–1.462)	1	0.362	1.2	0.23
AA + GG vs AG	Super-dominant model	0.00	0.954	0.91 (0.74–1.12)	0.929 (0.764–1.130)	1.4	0.234	0.452	0.75

Note: * indicates that the 95% CI did not report 0, which proved that the combined effect of the indicators was statistically significant.

The overall results showed that the rs231775 locus polymorphism in the CTLA-4 gene was statistically correlated with the susceptibility to GD in children in terms of allele (A vs G): OR = 0.75, 95% CI (0.66–0.86); co-dominant model (GG vs AA): OR = 1.34, 95% CI (1.04–1.73); co-dominant model (AG vs AA): OR = 1.32, 95% CI (1.02–1.10); dominant model (AG + GG vs AA): OR = 3.81, 95% CI (2.17–6.70); and recessive model (GG vs AA + AG): OR = 1.23, 95% CI (1.05–1.45). The results are shown in Table 2.

**Figure 1:**
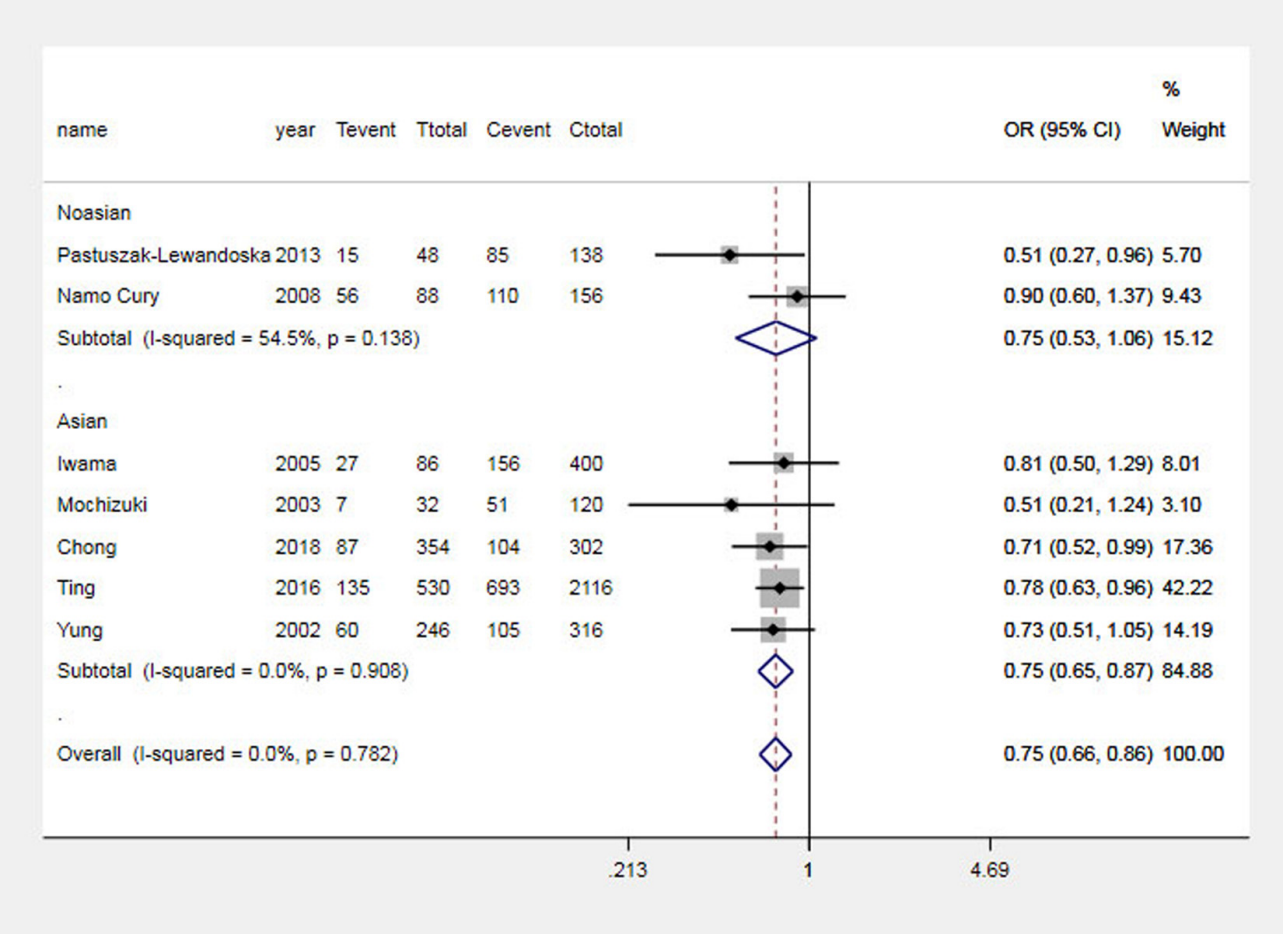
Meta-analysis forest plots (A vs G) of the correlation between rs231775 locus polymorphism of the CTLA-4 gene and GD in children. Tevent: Number of cases of this genotype in the experimental group; Ttotal: Total number of genotype cases in the experimental group; Cevent: Number of cases of this genotype in the control group; and Ctotal: Total genotype cases in the control group.

**Figure 2:**
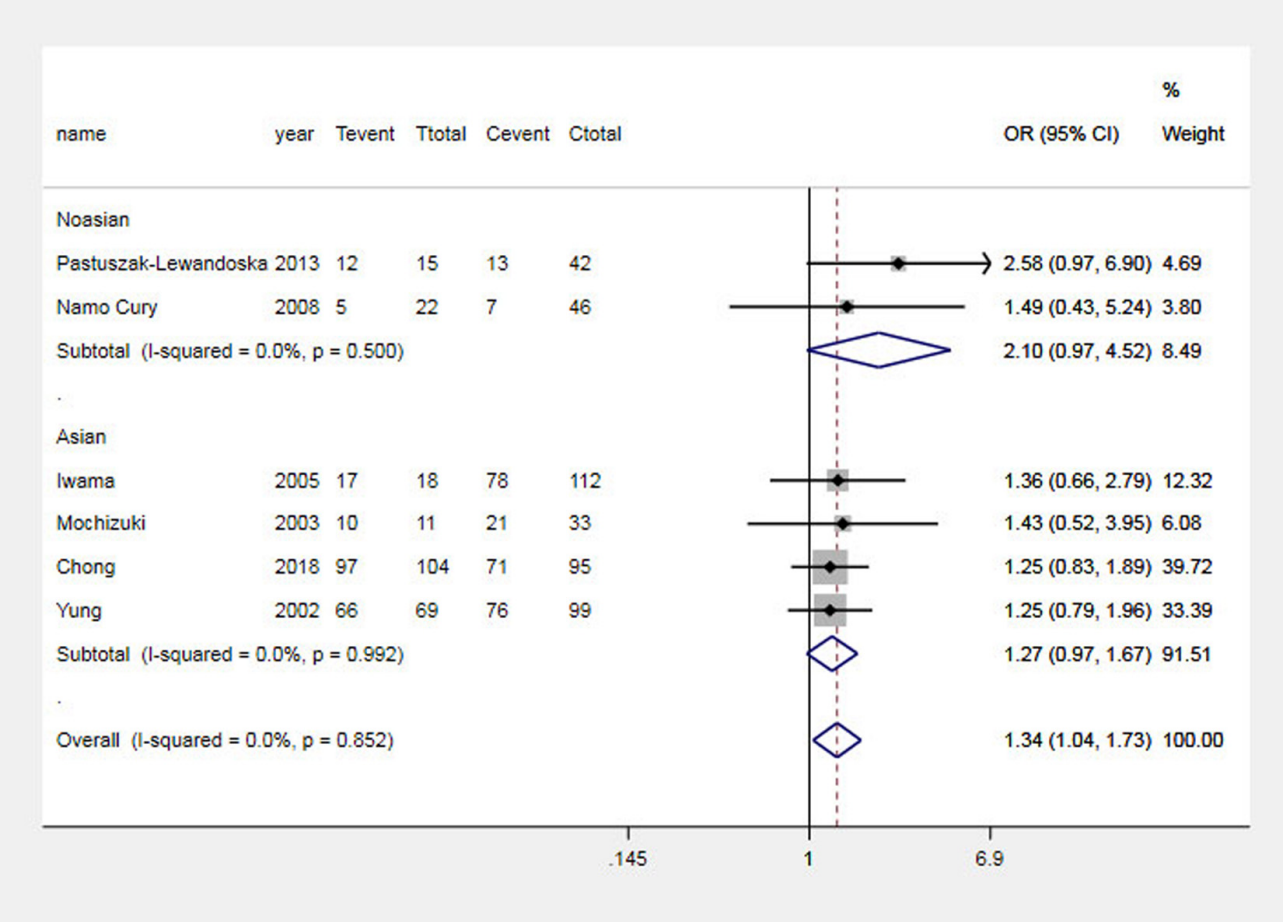
Meta-analysis forest plots (GG vs AA) of the correlation between rs231775 locus polymorphism of the CTLA-4 gene and GD in children. Tevent: Number of cases of this genotype in the experimental group; Ttotal: Total number of genotype cases in the experimental group; Cevent: Number of cases of this genotype in the control group; and Ctotal: Total genotype cases in the control group.

**Figure 3:**
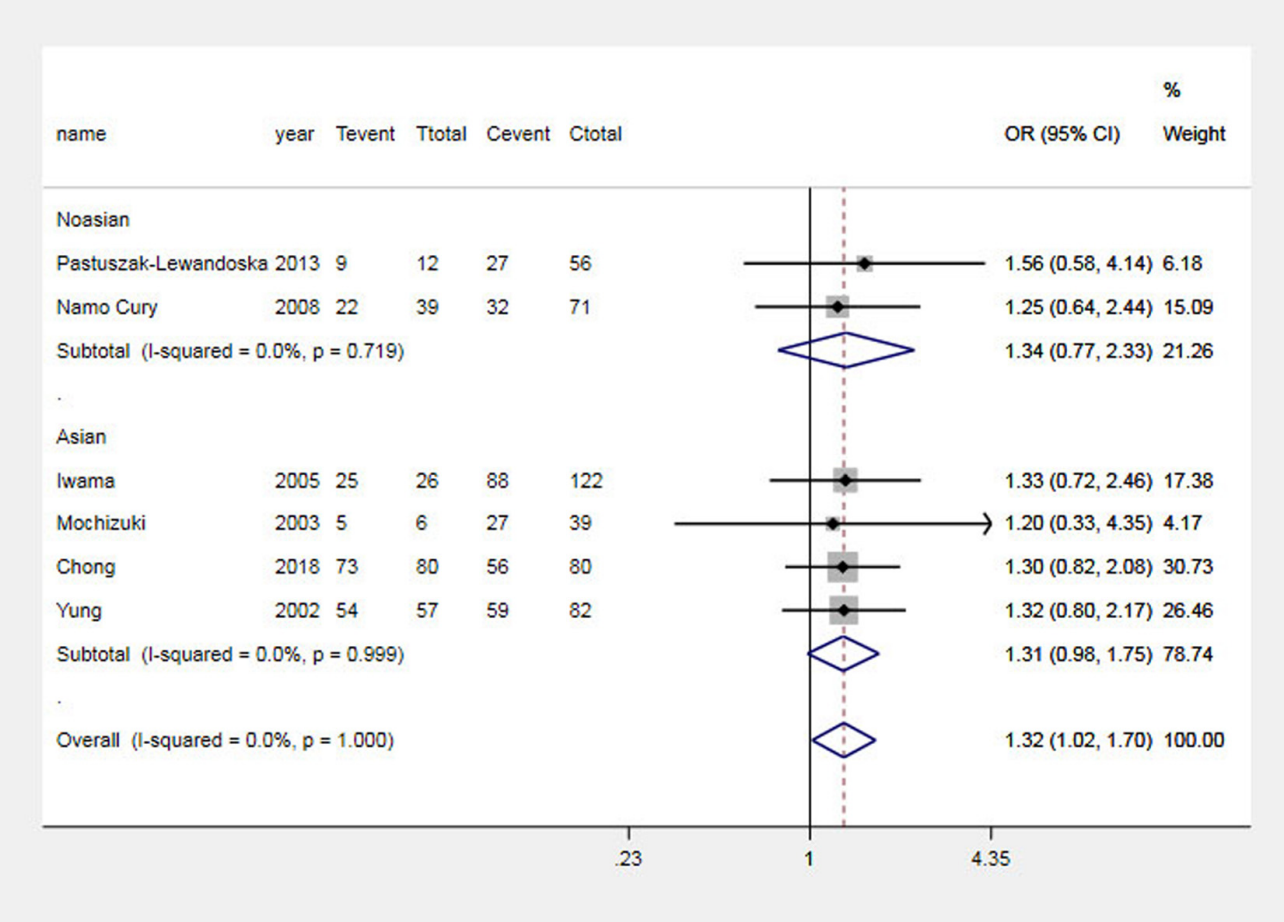
Meta-analysis forest plots (AG vs AA) of the correlation between rs231775 locus polymorphism of the CTLA-4 gene and GD in children. Tevent: Number of cases of this genotype in the experimental group; Ttotal: Total number of genotype cases in the experimental group; Cevent: Number of cases of this genotype in the control group; and Ctotal: Total genotype cases in the control group.

**Figure 4:**
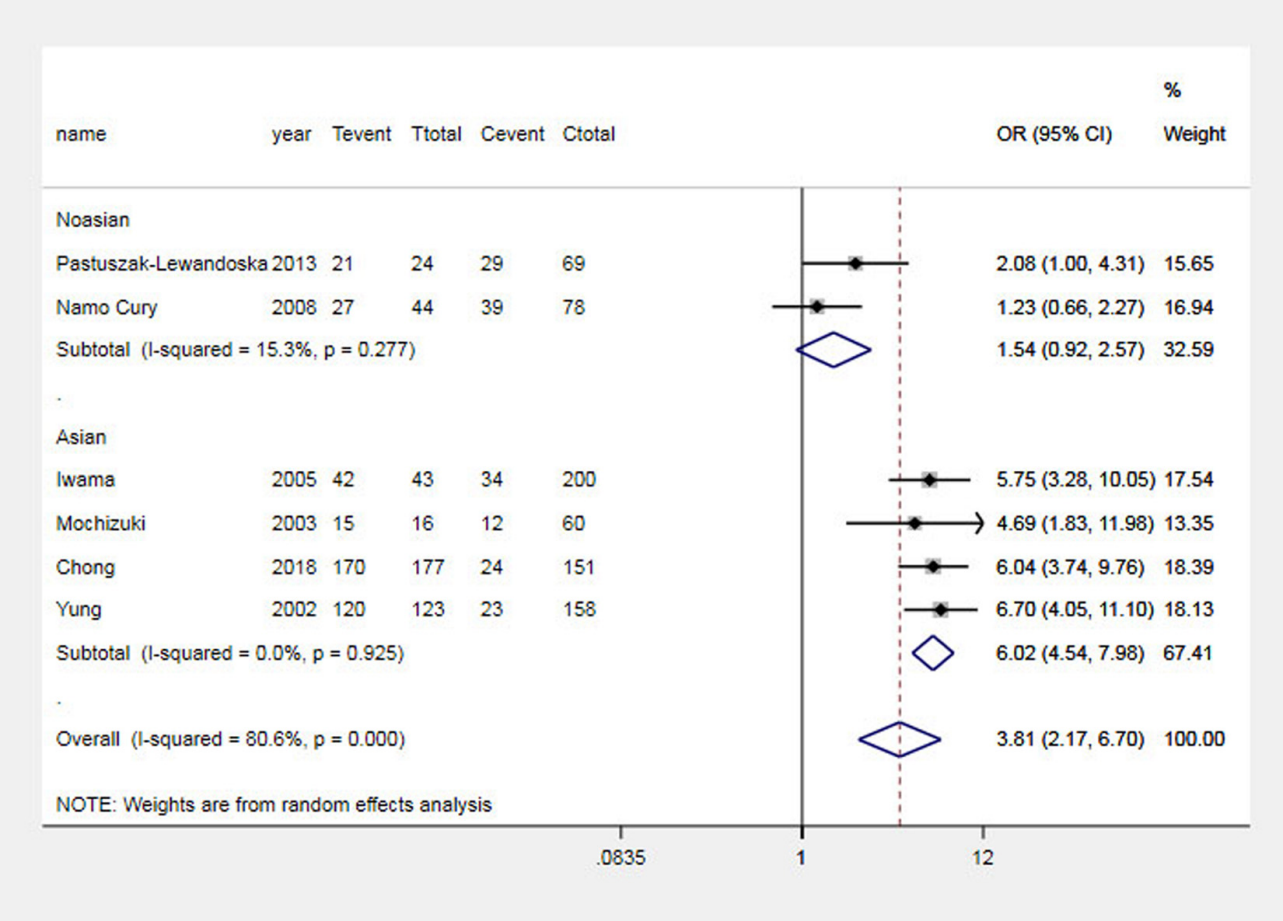
Meta-analysis forest plots (AG + GG vs AA) of the correlation between rs231775 locus polymorphism of the CTLA-4 gene and GD in children. Tevent: Number of cases of this genotype in the experimental group; Ttotal: Total number of genotype cases in the experimental group; Cevent: Number of cases of this genotype in the control group; and Ctotal: Total genotype cases in the control group.

The results of the analysis suggested that the G allele significantly increased the risk of GD in the Asian population and the total population, which was analyzed by comparing the G and A alleles of the CTLA-4 + 49 G allele, as shown in Figure 1. Carriers of the GG genotype have a higher risk of GD, as shown in Figure 2. In contrast, in Asian and general populations, carriers of the AG genotype have a higher risk of Graves’ disease compared to those carrying the AA genotype, which is shown in Figure 3. The AG + GG genotype is a risk factor for GD compared to the AA genotype, which is shown in Figure 4. The results of the recessive model analysis showed that CTLA-4 + 49 GG is a risk factor for GD (compared to the genotype AG + AA) in Asia and the total population. However, no significant differences were observed between the two populations of Caucasians mentioned above, as shown in Figure S2.

**Figure 5:**
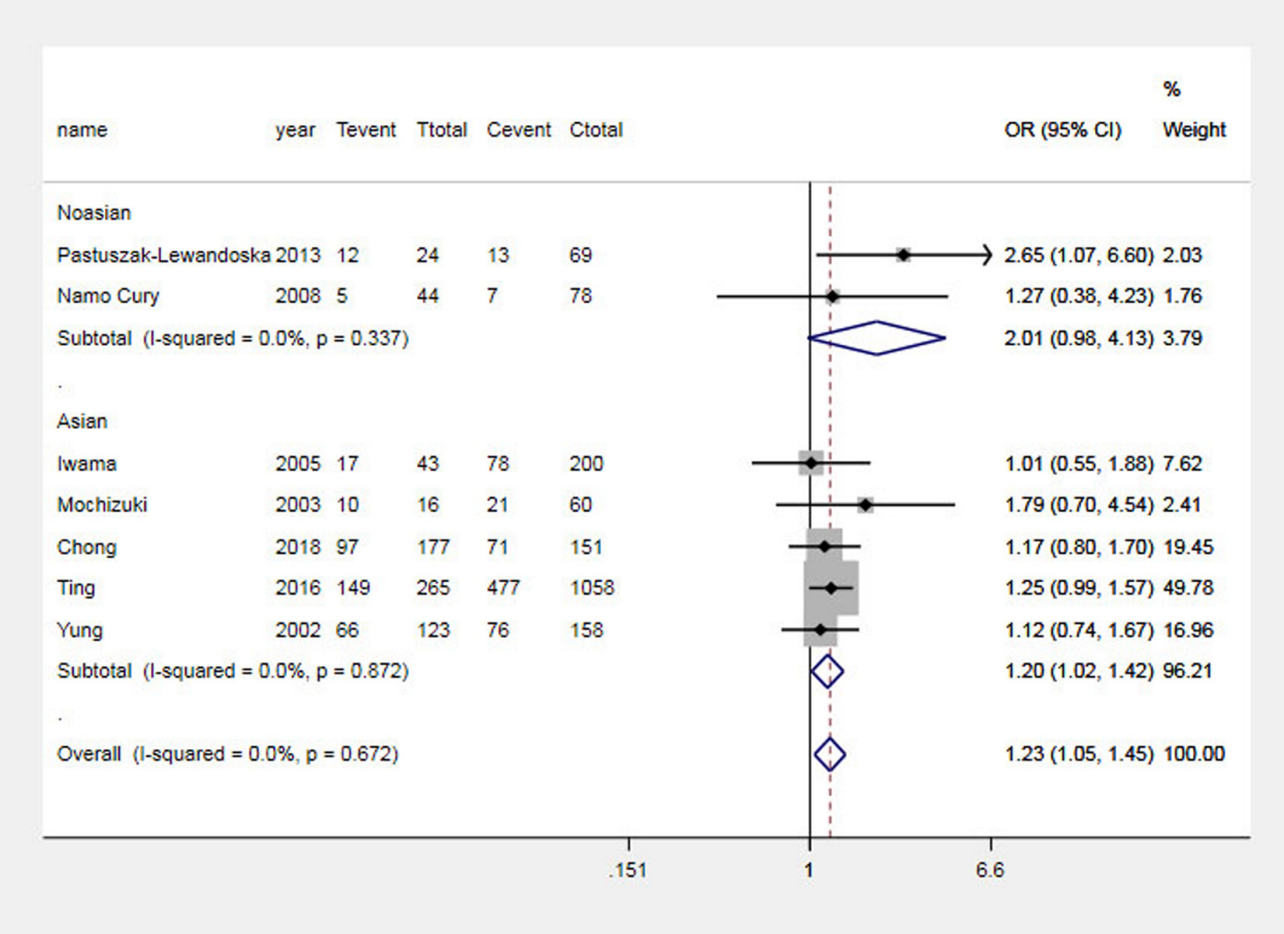
Meta-analysis forest plots (GG vs AA + AG) of the correlation between rs231775 locus polymorphism of the CTLA-4 gene and GD in children. Tevent: Number of cases of this genotype in the experimental group; Ttotal: Total number of genotype cases in the experimental group; Cevent: Number of cases of this genotype in the control group; and Ctotal: Total genotype cases in the control group.

Additionally, no correlation was found between GD susceptibility and CTLA-4 + 49 locus polymorphism under the super dominant model AA + GG vs AG (OR = 0.91, 95% CI (0.74–1.12), which is shown in Figure 5.

### 3.4 Sensitivity analysis

Yanagawa et al. [6] found a significant impact on the combined results of the GG vs AA, GA vs AA, AG + GG vs AA, and GG + AA vs AG models. Therefore, our study excluded the above models. After elimination, the other studies showed a minor impact on the results, indicating stability in the combined results of the above models. No study showed a significant impact on the combined results of A vs G and GG vs AG + AA models, indicating stable results, which are shown in Figure S3.

### 3.5 Publication bias test

The *P* values of Begg’s and Egger’s test results were between 0.230 and 0.848, with *P* ≥ 0.05. The trim and fill analyses showed that the OR value and 95% CI of each gene model showed no change before and after this method. As shown in Table 2, the conclusion was consistent, and the difference was not statistically significant. As shown in Figure S4, the funnel diagrams were symmetric, suggesting no publication bias.

## 4 Discussion

The decrease in CTLA-4 expression on the cell surface and its functional stability is believed to increase T-cell activation, which can lead to the development of autoimmune diseases. In the past 10 years, studies at home and abroad have shown that [14] CTLA-4 is a highly polymorphic gene. One of the loci that have the highest correlation with autoimmune thyroid diseases is the single nucleotide polymorphism at A/G 49 of codon 17 in exon 1. Furthermore, regional and ethnic differences were observed in the correlation analysis [15,16]. In an investigation on childhood-onset of GD, the presence of at least one G allele was shown to be associated with an increased risk of GD among Chinese children. Saika Iwama and others found that the CTLA-4 GG + AG genotype was significantly more frequent, and the AA genotype was less prevalent in Japanese GD patients with childhood onset than in controls [4,17,18]. Yung et al. [12] and others believed that CTLA-4 + 49 A/G mutation increased the risk of GD in children. However, Brazilian and Japanese scholars did not find any significant correlation between this mutation and children with GD [4,5], which was also verified in our study.

To perform the systematic meta-analysis, we selected case-control study exploring the correlation between CTLA-4 + 49 A/G polymorphism and GD in children belonging to different regions. The difference was found to be statistically significant between the case and control groups (*P* < 0.01) in the entire population for the G allele and the genotypes GG, AG, and AG + GG of the CTLA-4 gene locus rs231775. The subgroup analysis, based on regions, showed that the frequency of the G allele and the genotypes GG, AG, and AG + GG in the case group was significantly higher than in the control group, with statistically significant results (*P* < 0.01). The rs231775 locus was not associated with GD in children of Caucasian populations, which proved regional differences between the polymorphism of the rs231775 locus of the CTLA-4 gene and the susceptibility to GD in children. The above results showed that the CTLA-4 + 49 A allele could reduce the risk of GD. Compared to the AA genotype, GG and AG genotypes might show an increased risk of GD in children. The G allele may be the risk factor for GD in children, while the A allele may be the protective factor.

This is the first meta-analysis study investigating the rs231775 locus polymorphism of the CTLA-4 gene and the susceptibility to GD in children, which is carried out by retrieving the entire database of the network. This study showed that the rs231775 locus might cause GD in children (mainly the Asian population). However, this locus is not related to GD in Caucasian children. One reason for this could be the inclusion of fewer Caucasian population studies in the literature, while another could be ethnic heterogeneity. Therefore, to avoid covering up its real effect, different populations should not be blindly combined when performing research and analysis. Also, this study has some limitations. First of all, meta-analysis is a descriptive secondary analysis that is based on published studies. The results of the meta-analysis may be affected by different genetic testing methods. Only a few studies were included. Different studies addressed heterogeneity, the study design of variables, and different statistical methods [19], which may be due to publication bias. Eventually, both of these may have affected the results of this meta-analysis. Therefore, the conclusions of this study need to be further confirmed by a large sample and multicenter randomised controlled trials (RCTs).

The interpretation of publication bias: (1) Egger’s test, also known as the linear regression method, is the normal standard deviation of a study included in the meta-analysis. The regression equation SND = A + Bx precision was established for SND and precision. If there is no publication bias, the intercept (A) of the regression line is 0. If the regression line does not pass through the origin, then the larger the intercept A, the higher the degree of bias. The detection result *P* < 0.05 means small study effects. (2) Begg’s test is also known as the rank correlation test. Its main idea is to test the correlation between standardized effects and effect variance based on Kendall’s tau rank correlation test, that is, to test the correlation between effects and sample size. Under the null hypothesis of no publication bias, standardized effects can be considered independent and identically distributed, and there is no correlation between standardized effects. The test results depend primarily on the *p*-value, and publication bias is generally believed to exist if *P* < 0.05. (3) Based on the hypothesis that publication bias causes funnel plot asymmetry, the cut-and-complement method uses the iterative method to estimate the number of missing studies, but its significance is not to estimate the specific number of missing studies. After adding a part of the studies, a meta-analysis will be conducted again. If the estimated value of the combined effect size does not change significantly, it indicates that publication bias has little influence, and the result is relatively robust. In this study, the *P* values of Begg’s and Egger’s test results were between 0.230 and 0.848, *P* ≥ 0.05. The results of the shear and complement analysis showed that the OR values and the 95% CI of each gene model were basically unchanged before and after the shear and complement analysis, and the conclusions were consistent, indicating that the differences were not statistically significant [20,21].

Existing studies have shown that the CTLA-4 gene polymorphism is closely correlated with complications, drug effect, and GD recurrence [16]. The RCTs are expected to collect data on the above three aspects in the future to further confirm the correlation between them.

## Supplementary Material

Supplementary Figure
