# Supplementary material for: Meta-analysis of the rs231775 locus polymorphism in the CTLA-4 gene and the susceptibility to Graves’ disease in children
Source: Open Life Sci. 2023 Apr 28;18(1):20220589. doi: 10.1515/biol-2022-0589 (PMC10148602; doi:10.1515/biol-2022-0589)
Supplement: Supplementary Figure [file biol-2022-0589-sm.pdf]

Supplementary material

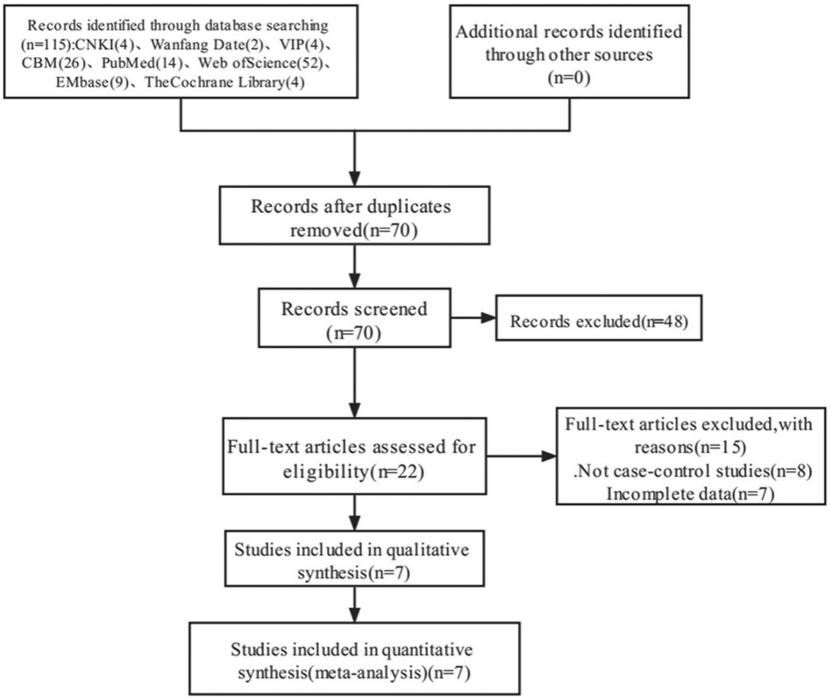

Figure S1: The process and results of the literature screening.

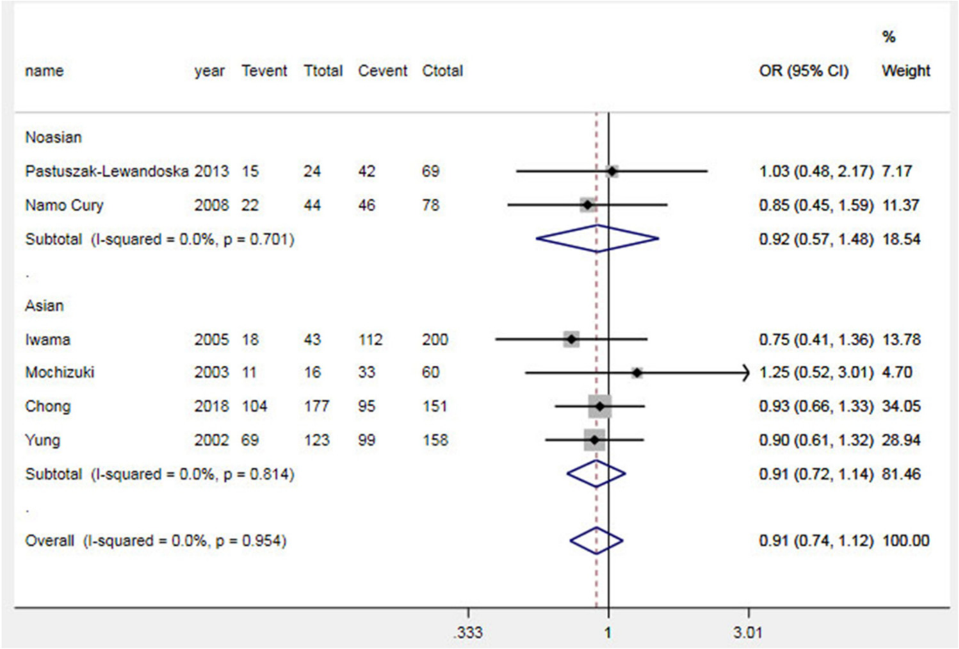

Figure S2: The meta-analysis forest plots (AA+GG vs AG) of the correlation between rs231775 locus polymorphism of the CTLA-4 gene and graves disease in children.

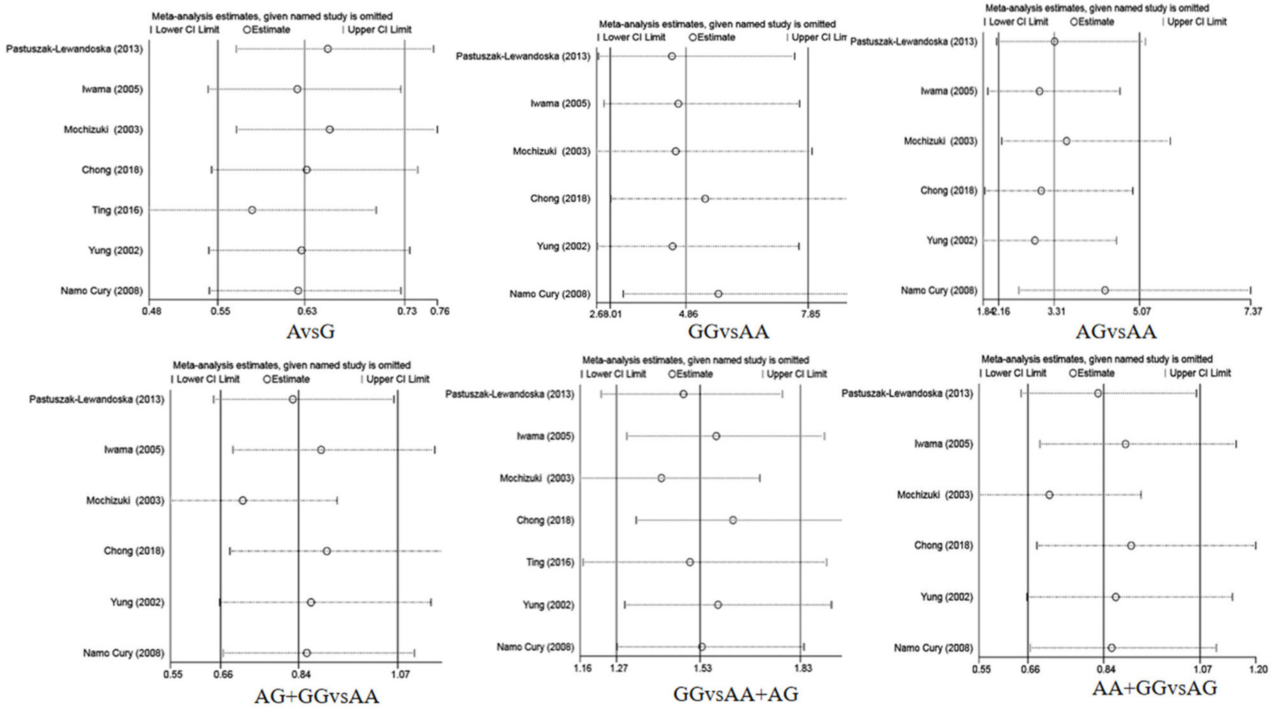

Figure S3: Sensitivity analysis of the correlation between rs231775 locus polymorphism of the CTLA-4 gene and graves disease in children.

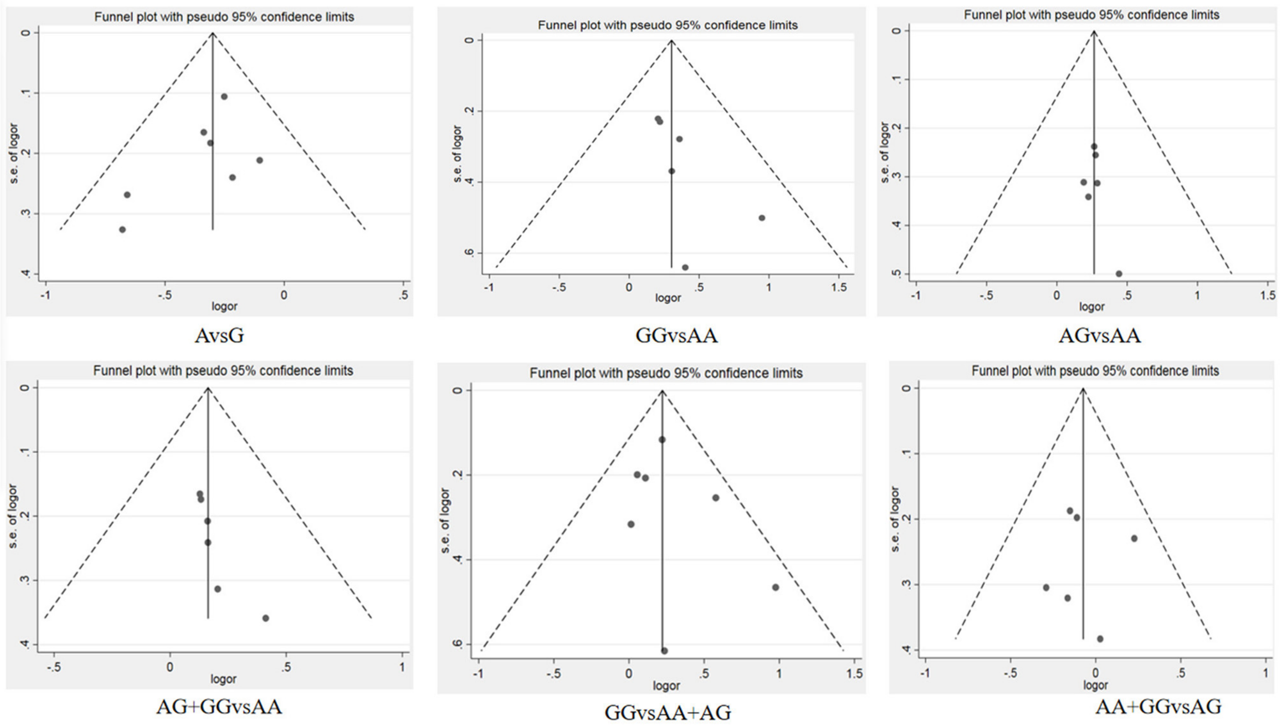

Figure S4: The Funnel diagrams of bias analysis exploring the correlation between rs231775 gene polymorphism and graves disease in children belonging to different regions.
